# Supplementary figures and images for: Validation of an orthotopic non-small cell lung cancer mouse model, with left or right tumor growths, to use in conformal radiotherapy studies
Source: PLoS One. 2023 Apr 13;18(4):e0284282. doi: 10.1371/journal.pone.0284282 (PMC10101527; doi:10.1371/journal.pone.0284282)

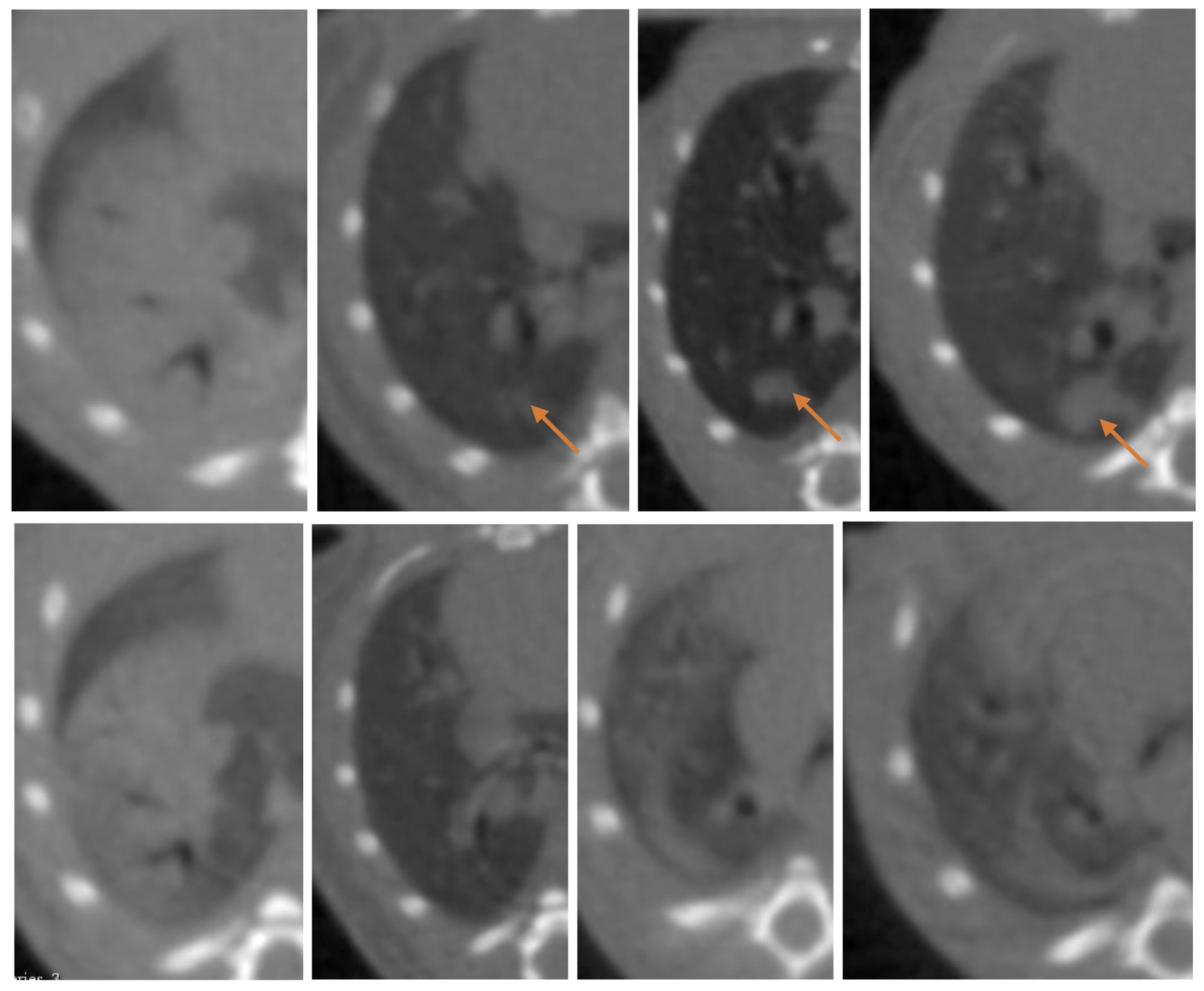

Supplement: S1 Fig — Each column represents a different time point, from the left the time points were post-op (far left column), imaging at first lesion which was two weeks after surgery (2nd column from the left), 3) two weeks after first lesion (2nd column from the right), and 4) one week after previous time point (far most right column). The presence of the H1299 lesion is indicated by an orange arrow. (TIF) [file pone.0284282.s001.tif]

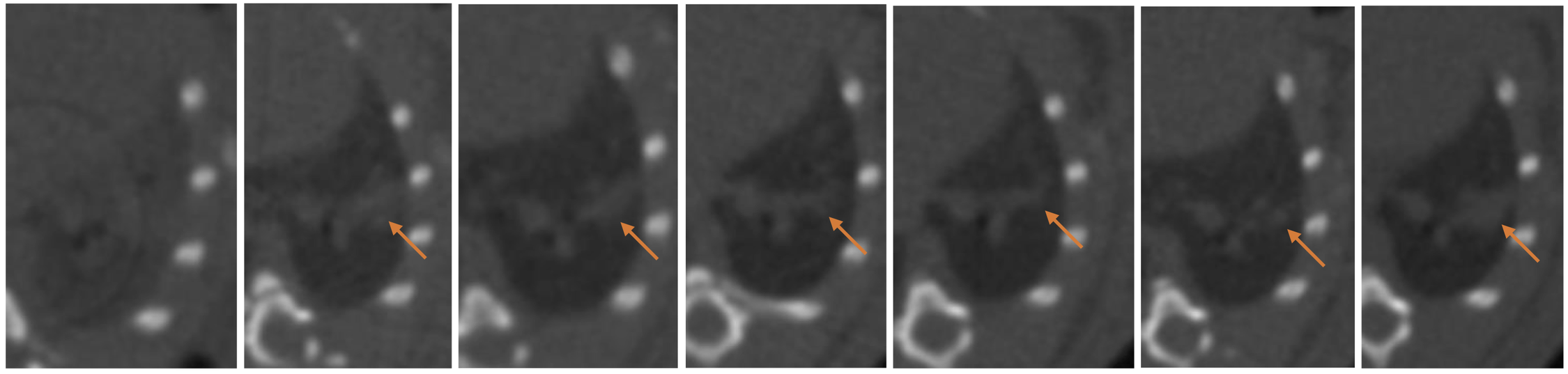

Supplement: S2 Fig — (TIF) [file pone.0284282.s002.tif]

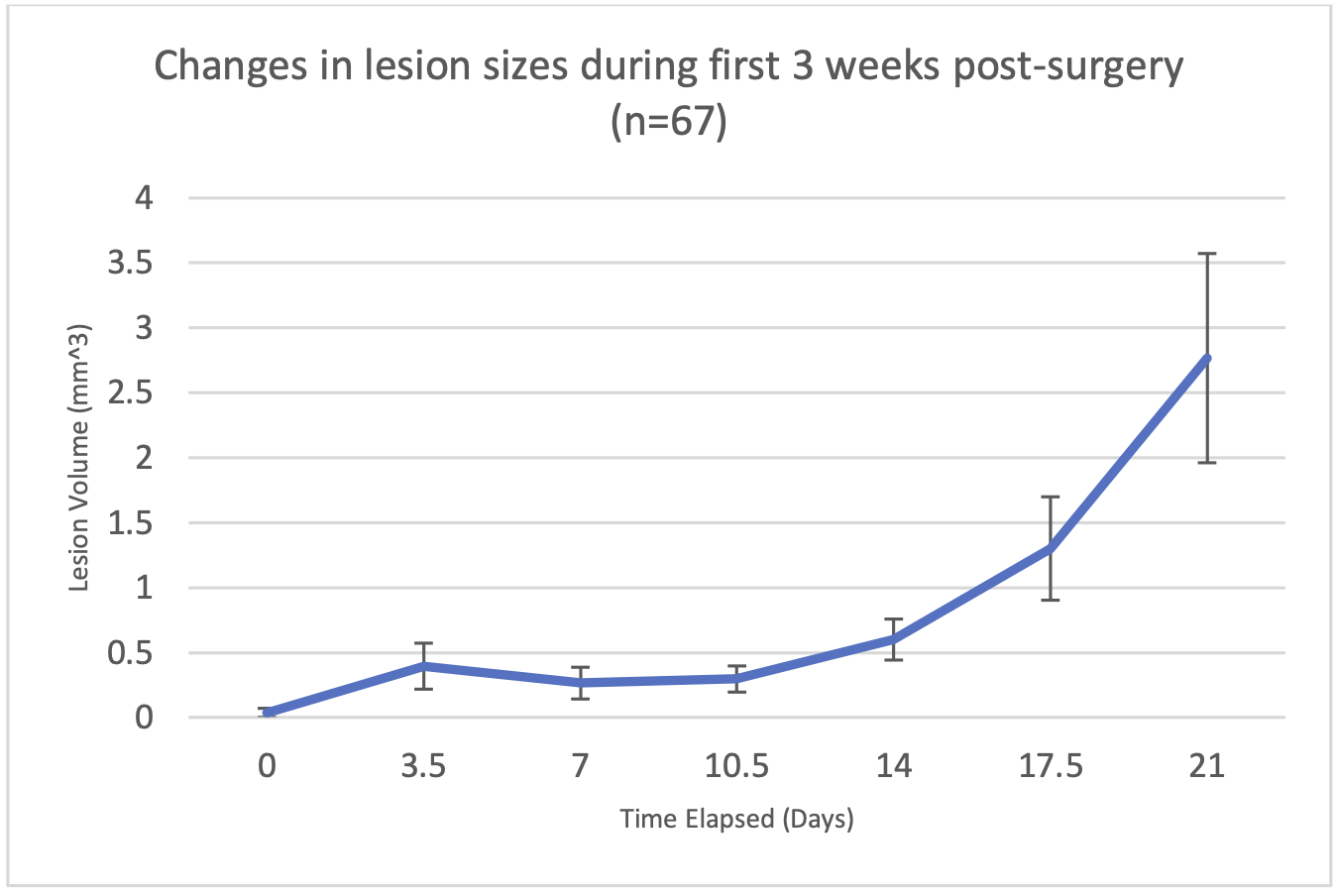

Supplement: S3 Fig — A stagnation of lesion growth occurs at the end of the first week in our population of 67 recorded surgeries. Post 2 weeks, the lesions are identified as tumors as they can be traced back from instances of confirmed tumor imaging retroactively. Error bars are indicative of standard error. (TIF) [file pone.0284282.s003.tif]

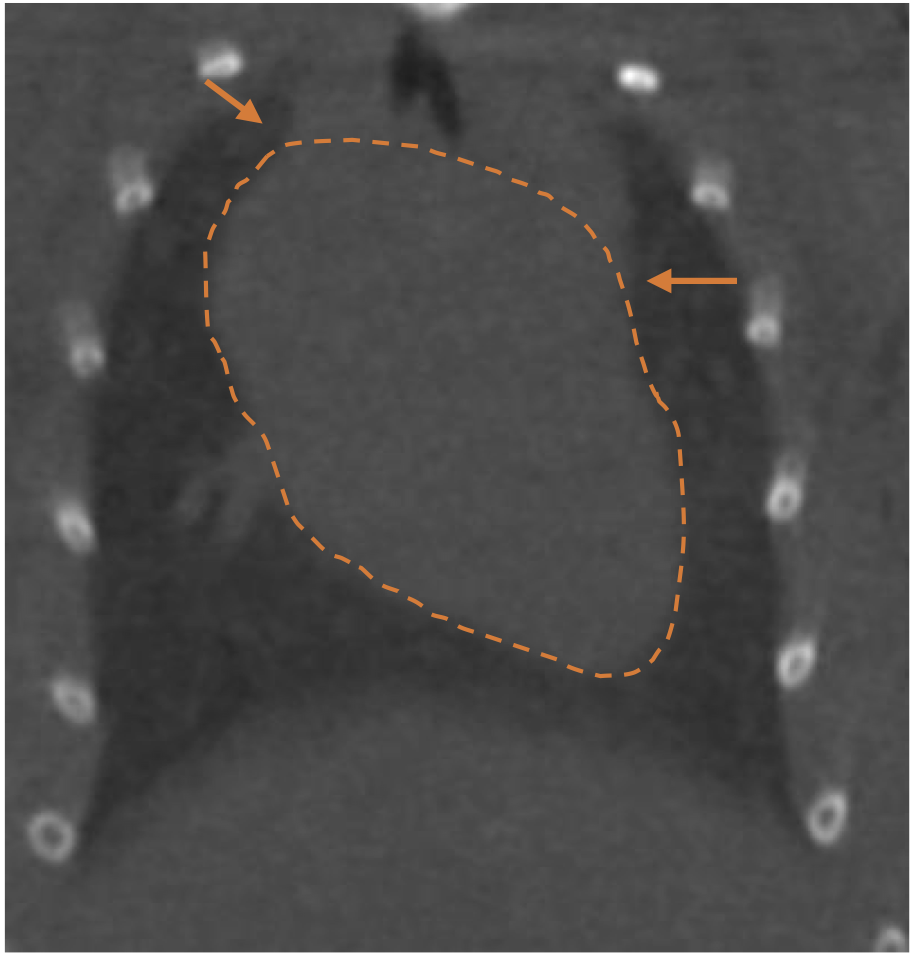

Supplement: S4 Fig — The delineated boundary between the heart and thymus was based an approximation of the heart shape as densities between the heart and thymus cannot be visually differentiated. (TIF) [file pone.0284282.s004.tif]

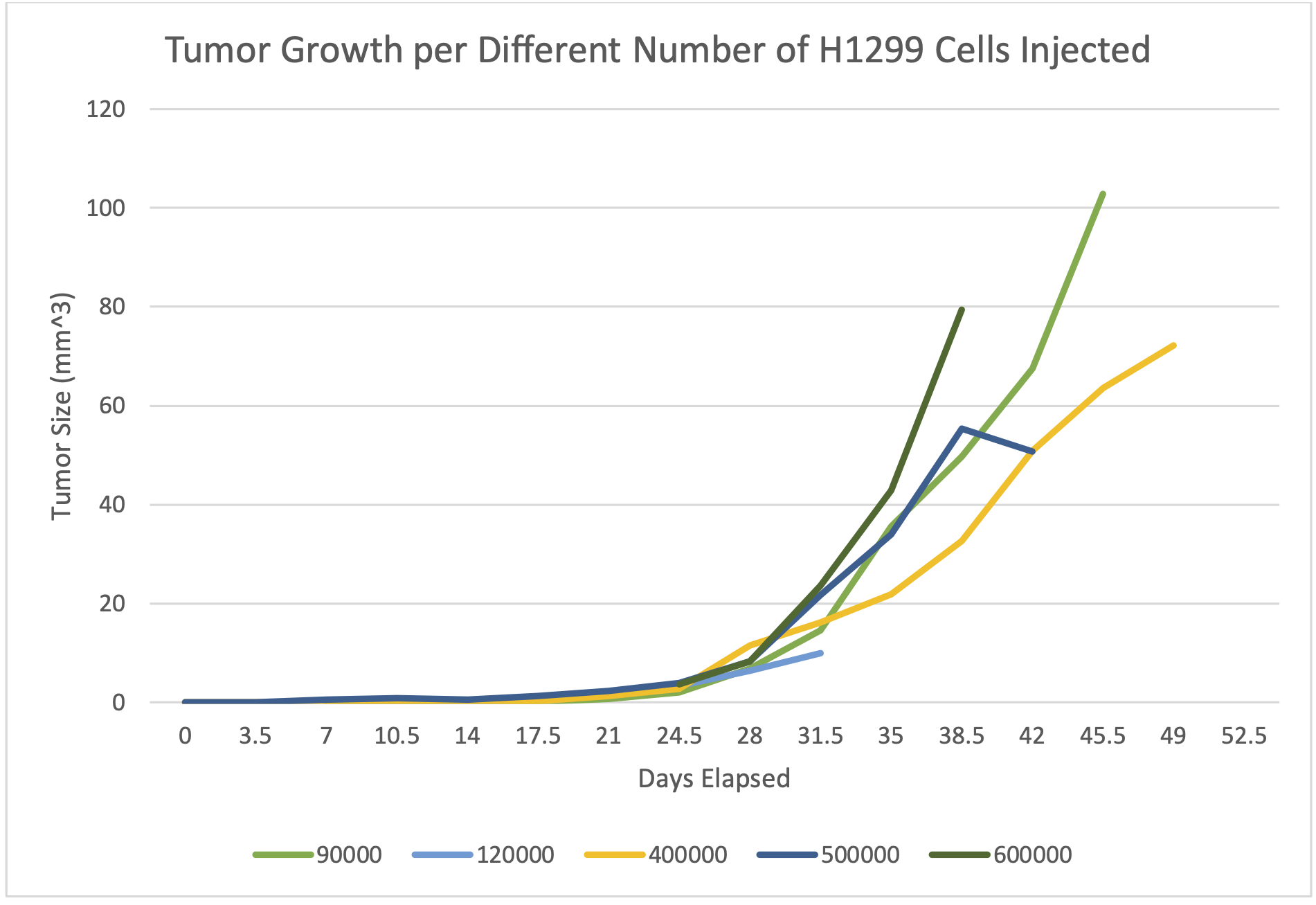

Supplement: S5 Fig — The tumors depicted in this plot were all right lung tumor models and were developed within 5 different animals. There was no correlation between increased number of injected tumor cells and increased tumor growth. (TIF) [file pone.0284282.s005.tif]
