# Supplementary material for: Validation of an orthotopic non-small cell lung cancer mouse model, with left or right tumor growths, to use in conformal radiotherapy studies
Source: PLoS One. 2023 Apr 13;18(4):e0284282. doi: 10.1371/journal.pone.0284282 (PMC10101527; doi:10.1371/journal.pone.0284282)
Supplement: Attachment — Submitted filename: PLOSReviewerRebuttal.docx [file pone.0284282.s006.docx]

PONE-D-22-27036

Validation of an orthotopic non-small cell lung cancer mouse model, with left or right tumor growths, to use in conformal radiotherapy studies

PLOS ONE

Dear Dr. Wang and Reviewers,

Thank you very much for all the time and effort in reviewing the manuscript and providing to us your comments and suggestions. They are very much appreciated. Below, in point form, we’ve added rebuttals to points brought about by reviewers:

Reviewer 1:

1. We’ve replaced the images with higher resolution ones. We attempted a schematic drawing however, we found that a schematic, when not overlayed onto an image, to be equally as difficult to decipher. We ultimately added the original anatomical landmark labelling to the new updated images. We find that this is the clearest representation and labelling of anatomical landmarks.
2. We’ve enlarged the arrows and red lines so that they are more visible against the background CT imaging.
3. In this case it does not. Omnipaque functions as an iodine based radiocontrast agent which enhances visibility of structures which uptake the contrast agent. Omnipaque is also a radiocontrast agent commonly used in the clinic and as such is designed to not adversely affect image quality. Matrigel is an extracellular matrix (ECM) preparation which solidifies at body temperature and provides an appropriate surrounding for the cancer cells to grow. Neither of these agents affect CT resolution which is based on the size and number of detector elements, the size of the X-ray focal spot, and the source-object-detector distances. The latter two of these three characteristics have been optimized as a part of regular equipment maintenance of the small animal irradiator system outlined in our protocol, while the first of these components does not change. Furthermore, we recognize that it could be plausible that the agents themselves contribute to imaging artifacts which affect the quality of the captured images. However, neither agent used in our protocol contain metals with high atomic numbers and, as such, will not cause possible artifacts which affect image quality.
4. Days elapsed refers to number of days since initial tumor implantation. We have made additions to the figure caption and to the figure axis label for Figure 7 to specify this information.
5. The two images do indeed show the same treatment regimen. A modification to Figure 8’s caption was made to highlight this.
6. Axis labels were added to the last graph in the figure. Graphs were also rearranged to ensure better alignment.

Review #2

1. Our intentions with the passage in the introduction were to highlight the limitations of bioluminescent imaging specifically when applied to studies involving external beam radiotherapy, such as the study of radiosensitizer responses or combined therapies on solid single nodule tumors common to NSCLC. In the context of these studies, treatment planning is dependent on clear 3D imaging reconstructions of tumors. We added an additional sentence to clarify this point. We agree with the reviewer that bioluminescence imaging does indeed have high detection sensitivity, but this property is disadvantageous in studies requiring tumor treatment planning as it is unable to clearly delineate exact tumor volumes. In our introduction, we were trying to highlight that CT imaging is the better method for applications involving external beam radiotherapy treatment planning in an orthotopic animal model. However, we felt that it was also important to highlighting the limitations of bioluminescence, which is a very popular and useful imaging method that has seen application in orthotopic models.

With regards to immune response, while we agree with the reviewer that studies featuring the adaptive immune responses, which features T-cell activity is not possible, as the athymic nude mice utilized in our model lacks T-cells, we do feel that these models can be used in studying innate immune responses. And while studies featuring the innate immune response and their response to transfected cells are limited, we wanted to still mention the possibility of interaction as we believe it is still valuable to be aware of possible innate immune responses even in adaptive immune compromised mice models. To that end we’ve added a sentence specifically clarifying that the effect most relevant for immunocompromised mice is effects to the innate immune system.

2. We have added additional clarifying statements regarding the limitation of CT imaging with regards to tumor size and dissemination. We agree the lack of verifying dissemination of cancer cells is a limitation to our imaging technique. Our protocol featuring CT imaging can only confirm the presence of successful injection into the lung parenchyma, it cannot confirm dissemination to other parts of the body. Furthermore, we noted that the identification of exceedingly small lesions can only be identified retroactively, requiring a series of follow-up imaging which provides a growth trajectory that can be followed back in time to identify the smallest possible lesion. While this is not the most effective way, this method of tumor identification was a recommendation resulting from our collaboration and consultation with a qualified clinical radiologist and replicates how clinicians approach diagnoses using CT imaging for lung lesions.
3. Human clinical protocols utilize much higher cumulative doses which aren’t relevant in mouse models and cannot be delivered by the small animal irradiation system. This is due to differences in dose rates and the treatment planning for small rodents being not as efficient or precise compared to clinical planning and delivery. As such it is not possible to extrapolate pre-clinical doses from clinical protocols and vice-versa. As an example, in clinical protocols for stereotactic body radiotherapy (SBRT) for the treatment of stage II non-small cell lung cancers (NSCLC), the prescribed dose is typically 20 Gy per fraction in 3 fractions resulting in a cumulative dose of 60 Gy. However, in clinical applications of SBRT the treatment of stage II NSCLC tumors are, proportionally to the lung, are very small. As such SBRT field sizes are also very small allowing for healthy lung tissue sparing. However, due to the size of the mice lung, irradiation fields often cover a majority of a single lung. As such there is not much tissue sparing. As such, we did not attempt to base our preclinical treatment protocol on that which is currently used in the clinic.

However, despite clinical protocols being difficult to directly adapt in a pre-clinical setting, there are pre-clinical studies that have been conducted which demonstrate that our applied 20 Gy dose is well tolerated in rodents. We’ve added the citations as well as a clarifying statement in the methods to explain that our choice of 20 Gy was based on previous studies which showed that there is good rodent tolerance for the dose.

4. Tumors remain visible if they are 1) located in the lung, 2) maintain a shape distinctly different from surrounding vessels and soft tissue structures and 3) can be interpreted over time (meaning there are more than just one static image at one time point and that changes in tumor can be appreciated and followed). Throughout our 30-day follow-up period the tumor remained visible unless macroscopic evidence is not observed. This is due to the tumor being a denser soft tissue density among the lower density lung parenchyma. As mentioned in the results, there was an overlap period between the disappearance of the initial high density, likely due to acute inflammation or residual contrast media, and the appearance of the lesion. This overlap makes it difficult to determine the smallest lesion. Despite this overlap we were able to detect lesions that were as small as 0.1 mm^3^ as can be appreciated on our plot in Figure 5.
5. This was a combination of CT and resection. An additional methods section was added to detail the process of visual validation at the time of animal euthanasia.
6. We’ve changed the phrasing to the external lung tumor ratio that were presented in our study with the exact ratios that we observed. We’ve also added mentioned of the findings of Jarry et al., specifically with regards to their observation of tumor growth outside the lung and observations of reduced deep penetration of implanted cancer cells.
7. As the reviewer had also alluded to in point 5, we have since added the much-needed section regarding animal euthanasia and identification of region of lesion growth. Tissues were not analyzed by histology, but visual identification was conducted to validate the results from the imaging section.
8. Due to the instances of lung external tumors and the application of conformal radiation treatments featuring external beams, it is difficult for us to ascertain the true effect of tumors on animal that are not visible by CT, such as the lung external tumors. Furthermore, our model is also limited in its capacity to detect metastasis. The combination of non-visible tumors and possible metastatic sites can affect animal survivor despite target tumor control. Further study on out of field tumors is warranted for an orthotopic model like ours. We have added a statement which relates our mention of lung external tumors to the outcomes seen in Figure 5 and Figure 8.

Editorial Points:

1. We have made a change to our financial disclosure. While we did include in the acknowledgements, we also added it to the cover letter. We understand that the financial disclosure is likely not appropriate for the acknowledgements, as was specified by the formatting documents, but we kept it there in case, as per tradition. We will remove it if necessary.
2. Formatting made to the manuscript to adhere to PLOS One’s formatting requirements
3. Reintegration of the Supplemental information into the manuscript document.
4. Uploads of the Supplemental figures separately.
